# Oligodendrogliomas, cyclin pathway after the introduction of the molecular 2021 World Health Organization (WHO) criteria

**DOI:** 10.1093/braincomms/fcaf380

**Published:** 2025-10-01

**Authors:** Maria Angeles Vaz-Salgado, Juan M Sepulveda, Julie Earl, Jacqueline Gutierrez, Yolanda Ruano, Hector Pian, Diana Cantero, Berta Segura-Collar, Ricardo Gargini, Aurelio Hernández-Laín

**Affiliations:** Medical Oncology Department, Ramón y Cajal University Hospital, Madrid 28034, Spain; Biomarkers and Personalized Approach to Cancer Group (BioPAC), Area 3, Ramón y Cajal Health Research Institute (IRYCIS), Madrid 28034, Spain; The Biomedical Research Network in Cancer (CIBERONC), Madrid 28029, Spain; Medical Oncology Department, 12 de Octubre University Hospital, Madrid 28041, Spain; Biomarkers and Personalized Approach to Cancer Group (BioPAC), Area 3, Ramón y Cajal Health Research Institute (IRYCIS), Madrid 28034, Spain; The Biomedical Research Network in Cancer (CIBERONC), Madrid 28029, Spain; Neuropathology Unit, Pathology Department, imas12 Research Institute, Hospital Universitario 12 de Octubre, Universidad Complutense de Madrid, Madrid 28041, Spain; Neuropathology Unit, Pathology Department, imas12 Research Institute, Hospital Universitario 12 de Octubre, Universidad Complutense de Madrid, Madrid 28041, Spain; Department of Pathology, Ramón y Cajal University Hospital, IRYCIS, Madrid 28034, Spain; Neuropathology Unit, Pathology Department, imas12 Research Institute, Hospital Universitario 12 de Octubre, Universidad Complutense de Madrid, Madrid 28041, Spain; Neuropathology Unit, Pathology Department, imas12 Research Institute, Hospital Universitario 12 de Octubre, Universidad Complutense de Madrid, Madrid 28041, Spain; Neuropathology Unit, Pathology Department, imas12 Research Institute, Hospital Universitario 12 de Octubre, Universidad Complutense de Madrid, Madrid 28041, Spain; Neuropathology Unit, Pathology Department, imas12 Research Institute, Hospital Universitario 12 de Octubre, Universidad Complutense de Madrid, Madrid 28041, Spain

**Keywords:** oligodendroglioma, cyclin pathway, p16, CDKN2A deletion, D1 cyclin

## Abstract

In 2016, the World Health Organization (WHO) classification introduced molecular criteria [isocitrate dehydrogenase (IDH) mutation and 1p/19q codeletion] for the diagnosis of oligodendroglioma. The cyclin pathway (cyclin-dependent kinase inhibitor 2A deletion/p16 expression) has been extensively studied in this tumour but was performed in series based only on histopathological criteria. This study analysed this pathway in oligodendrogliomas with isocitrate dehydrogenase (IDH) mutation and 1p/19q codeletion. Cases with morphological diagnosis of oligodendroglioma were identified (182 cases). This same cohort was subsequently reclassified according to the current 2021 World Health Organization (WHO) (91 cases). The type of isocitrate dehydrogenase (IDH) (determined by high-resolution melting and immunohistochemistry) and telomerase reverse transcriptase (TERT) mutations were analysed. Also, p16, pRb and cyclin D1 were studied by immunohistochemistry. In the reclassified cohort, 82 cases (90.1%) had isocitrate dehydrogenase1 (IDH1) mutation and 9 cases (9.9%) had isocitrate dehydrogenase 2 (IDH2) mutation. Eighty cases (87.9%) had TERT mutation, 33 cases (36.3%) had 250T, and 47 cases (51.6%) had C228T mutation. 16 of 71 cases (22.5%) had no p16 expression, and was significantly associated with a worse prognosis; the median OS in the absence of p16 was 9 years (95% CI 9.36–17.35) versus 13.35 years with p16 expression (95% CI 9.36–17.35) *P* = 0.023 HR 0.41. With a clear difference in Grade 3 (4.76 versus 17.49 years). In a multivariate analysis, only the absence of p16 showed a statistically significant prognostic value (*P* = 0.034). In Grade 2 oligodendrogliomas, high cyclin D1 expression was associated with worse survival. In this cohort of oligodendroglial tumours classified by molecular criteria, the loss of p16 expression is associated with a poor prognosis, particularly in Grade 3 oligodendrogliomas.

## Teaser

Vaz-Salgado MA *et al.* analysed the cyclin pathway, especially p16 expression, in a retrospective cohort of 91 patients diagnosed with oligodendroglioma by the World Health Organization (WHO) 2021 criteria [isocitrate dehydrogenase (IDH) mutation and 1p/19q codeletion]. It confirms that the absence of p16 expression is associated with a statistically worse prognosis.

## Graphical Abstract

**Graphical Abstract:**
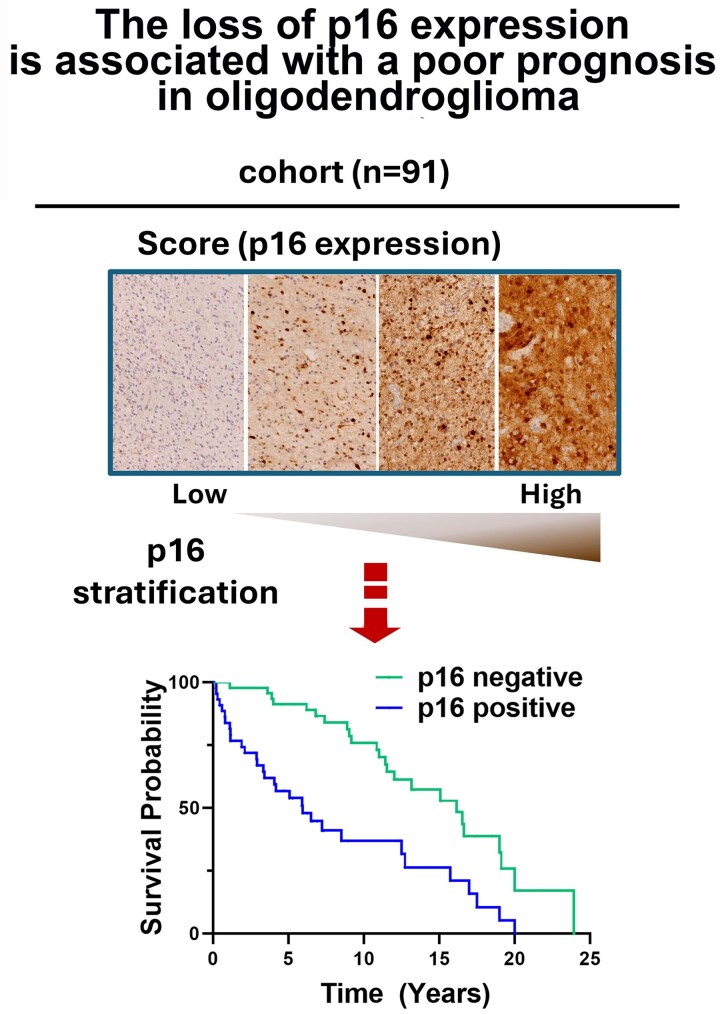

## Introduction

Oligodendroglial tumours are rare primary brain tumours, classically classified into Grade 2 and Grade 3, and affect young people.^1^

Often these tumours have an indolent behaviour but are usually incurable despite the extensive therapeutic effort. They have been considered typically chemosensitive, however, after progression, there is no standard of care, and even more, there has been no new systemic therapy despite the important molecular knowledge developed in recent years^2-4^ Therefore, new strategies are needed to improve survival in these patients.

In 2016, for the first time, the World Health Organization (WHO) classification of the Central Nervous system uses molecular criteria in addition to histology to define the different types of CNS tumours,^5,6^ and subsequently included in the 5th edition of 2021.

In the case of oligodendroglioma, according to the current 2021 WHO classification, the diagnosis requires IDH mutation and 1p/19q codeletion.

The cyclin-dependent kinase inhibitor 2A (CDKN2A) gene is a tumour suppressor gene located at chromosome 9, locus p21.3, that encodes the p14ARF and p16INK4a proteins. Both proteins are generated by alternative splicing of the mRNA from CDKN2A gen.^7^ CDKN2A is the second most altered tumour suppressor gene in cancers, including gliomas^8^ and its inactivation promotes the G1-S phase transition. The most frequent mechanism of CDKN2A molecular inactivation is homozygous deletion, but molecular inactivation can also be a result of promoter hypermethylation, missense mutations or post-transcriptional regulation mechanisms.

For the reason that the prognostic impact of CDKN2A homozygous deletion is crucial, the last WHO classification of central nervous system tumours integrated this deletion into the grading of this type of glioma.^6^ Homozygous deletion of CDKN2 can be detected by various techniques, such as fluorescence *in situ* hybridization, comparative genomic hybridization array or potentially next generation sequencing. All these methods are complex, time-consuming and may result in delayed diagnosis and subsequent treatment. IHC is not currently recommended as a reliable surrogate test. However, there is a p16-protein-targeting antibody, which is used in daily practice to diagnose other diseases. Taken together, we decided to focus our study on the clinical implication of p16 expression measured by IHC in oligodendroglioma.

In addition, many of the studies of the cyclin pathway have been carried out in a series of patients with oligodendrogliomas diagnosed by histopathological criteria. Information on alterations in the cyclin pathway and the prognostic implication is less well studied in oligodendroglioma series that are better characterized at the molecular level (with both IDH mutation and 1p/19q codeletion), following the diagnosis criteria established by the WHO 2021.

On the other hand, The TERTp mutation is commonly found in glioblastomas GBM at a prevalence rate of 70–90% and in oligodendrogliomas at 78–100%.^4^ The high incidence of TERTp mutations in both highlights the importance of this mutation in glioma biology.^9^ However, while in glioblastomas is associated with a worse prognosis, oligodendrogliomas tend to have a more favourable prognosis when they present the TERTp mutation,^9^ encompassing the two most common mutations in TERT (about 90% of total cases), C228T and C250T in their promoter region.

In this work, we analyse some of the most important molecular alterations in this tumour, such as IDH, TERT and also the cyclin pathway (p16, Cyclin D1 and pRb) in a series of oligodendrogliomas with IDH mutation and 1p/19q codeletion.

## Materials and methods

This a retrospective cohort study.

Cases of oligodendrogliomas were identified from registries in the pathology department over 37 years, between 1977 and 2014. A total of 182 cases were diagnosed with oligodendroglioma or oligoastrocytoma, Grade 2 or Grade 3, based on histological appearance. Oligodendroglioma tissue samples, derived from paraffin-embedded tissue, were obtained after written informed consent from the patients and with the approval of the Ethics Committees of the 12 de Octubre University Hospital (CEI 19/569) and in accordance with the Declaration of Helsinki.

These cases were reclassified with actual 2021 WHO criteria (that includes IDH mutation and 1p/19q codeletion).

IDH mutations were determined by high-resolution melting analysis with complementary immunohistochemistry (IHC), and the codeletion of 1p/19 q was performed by fluorescence *in situ* hybridization. TERT mutations were also analysed by pyrosequencing.

The cyclin pathway was also studied in the reclassified cohort.

Mutations in the TERT gene were analysed, as well as p16, pRB, and Cyclin D1 by immunohistochemistry (cyclin pathway).

Rb was classified as positive or negative. Rb immunoreactivity was considered positive when more than 50% of tumour cell nuclei showed intense positive staining.

P16 was classified into six groups: no immunohistochemical expression, expression <10% in tumour cell nuclei, expression <25%, expression between 25 and 50%, expression between 50 and 75% and expression between 75 and 100%. The cut-off percentage for positivity was 1%.

Cyclin D1 was classified into six groups: no immunohistochemical expression, expression <10% of tumour cell nuclei, expression <25%, expression between 25 and 50%, expression between 50 and 75% and expression between 75 and 100%. The cut-off percentage for positivity was 1%.

Clinical data were obtained from all cases, regarding diagnosis, recurrence, treatments received and overall survival. All cases were followed up until March 2020 and censored at this point. To address the missing values, the elimination of those variables was done. The loss to follow-up was addressed with a phone call to all the cases.

The study has been approved by the local Ethics Committee and consent was obtained according to the Declaration of Helsinki.

### Statistical analyses

The statistical study was conducted using SPSS version 24.00. The association of dichotomous qualitative variables was evaluated within the study using the Chi-square test. For the survival study, Kaplan–Meier curves were used; univariate survival analysis was performed by comparing the different groups using the Log-Rank test. Finally, multivariate survival analysis was performed using Cox proportional hazards models. Statistical significance was set at *P* < 0.05. The raw data are available upon request from the corresponding author.

## Results

### Molecular characterization of the reclassified cohort

A total of 91 cases met the criteria of IDH mutation and 1p/19q codeletion. In this reclassified cohort, molecular analysis has been performed.

After the reclassification, all cases had IDH mutations, as this was one of the criteria included in the WHO 2021. An IDH1 mutation was found in 82 cases (90.1%) and an IDH2 mutation was found in 9 cases (9.9%). The 1p/19q codeletion was present in all cases of the reclassified series as it was a requirement to consider the patient as an oligodendroglioma. A TERT mutation was found in 80 cases (87.9%) and wild type in 4 cases (4.4%) and could not be assessed in 7 cases (7.7%) due to technical problems. A TERT mutation at C250 was found in 33 cases (36.3%), and a mutation at C228 was observed in 47 cases (51.6%) (Table 1).

**Table 1:** Frequency of IDH and TERT mutations

	IDH1 mutation %	IDH2 mutation %	TERT mutation %	TERT C250 %	TERT C228 %
Total cohort	90.1	9.9	88	36.3	51.6
Oligodendroglioma G2	88.1	11.9	83	37.3	45.8
Oligodendroglioma G3	93.8	6.3	96.5	34.4	62.5

After the reclassification, IDH mutations were present in all cases, with IDH1 mutation in 90.1% of cases and IDH2 mutations in 9.9%. The 1p/19q codeletion was present in all cases of the reclassified series and a TERT mutation was found in 87.9% of cases, of which 36.3% were C250 and 51.6% were C228.

No differences were observed in median OS according to IDH mutation type (IDH1 versus IDH2 mutations determined by high-resolution melting) or TERT mutation (wild type versus C250T versus C228T), neither in Grade 2 nor Grade 3 oligodendroglioma series.

### P16 expression (by IHC) in the overall cohort (including Grades 2 and 3)

**Figure 1:**
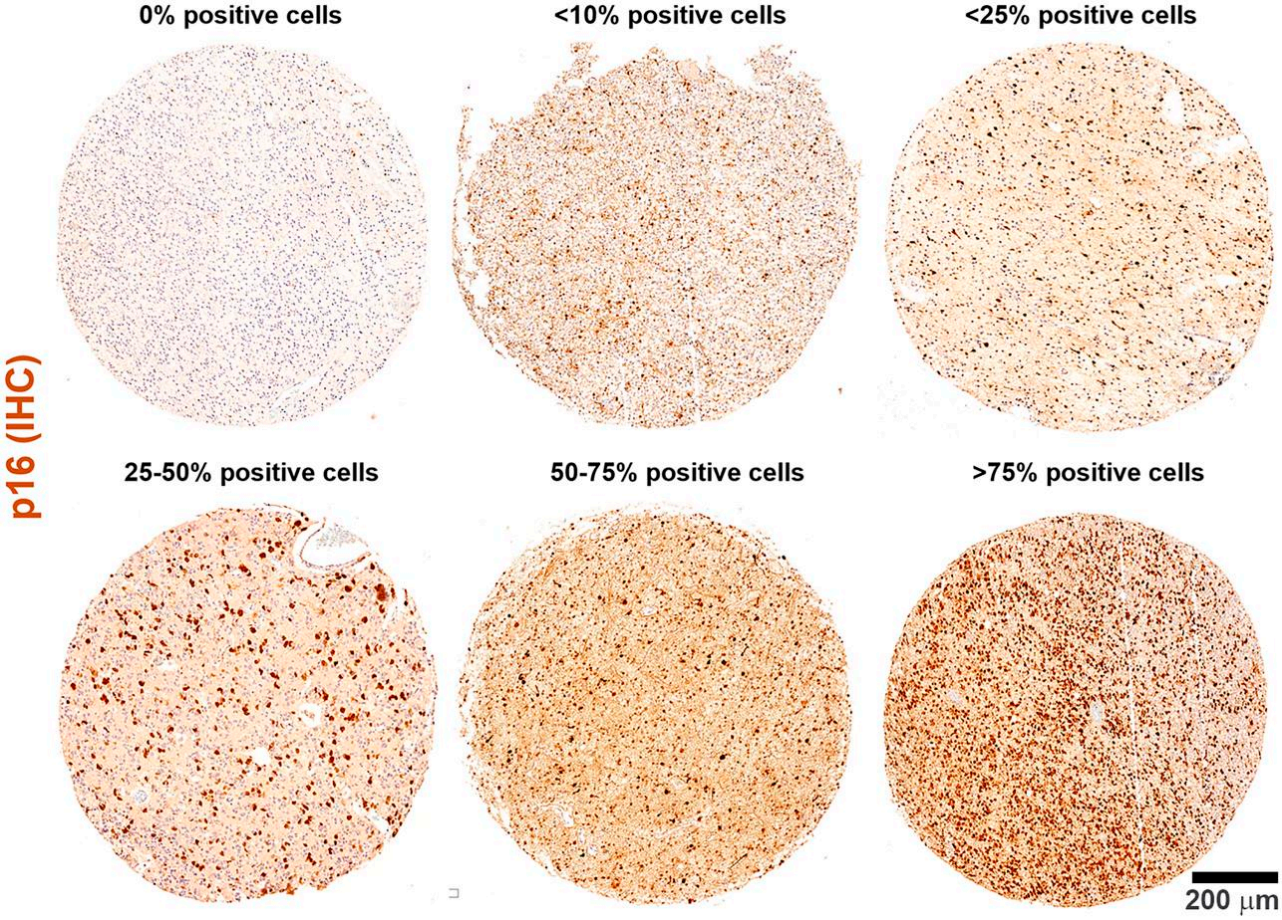
**Frequency of p16 expression.** p16 expression was absent in 22.5% of all cases analyzed (*N* = 16). The level of p16 expression <10% in 43.7% of cases (*N* = 31), 10–<25% in 23.9% (*N* = 17), 25–<50% in 7% (*N* = 5), 50–<75% in 1.4% (*N* = 1) and 75–100% in 1.4% (*N* = 1).

In the total series of 71 cases analysed, p16 expression was absent in 16 cases (22.5%). Regarding the level of expression of p16, it was <10% in 31 cases (43.7%), 10–<25% in 17 cases (23.9%), 25–<50% in 5 cases (7%), 50–<75% in 1 case (1.4%) and 75–100% in 1 case (1.4%) (Fig. 1).

**Figure 2:**
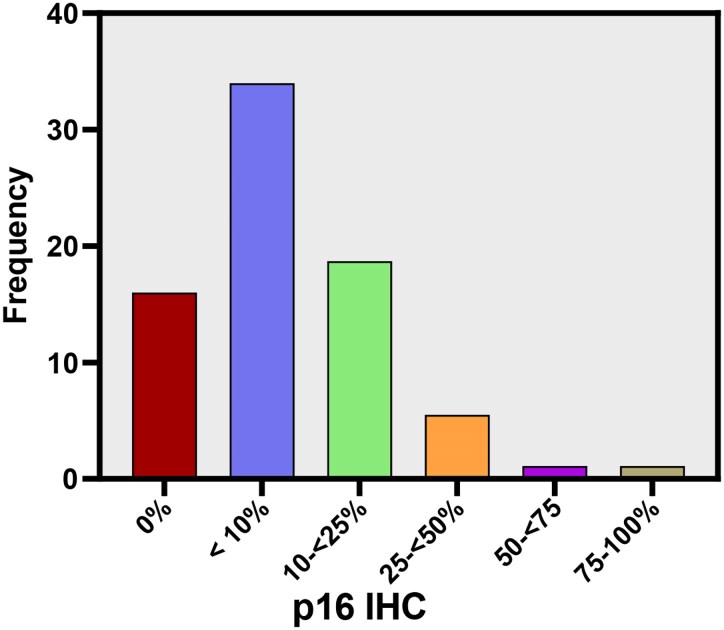
**Oligodendrogliomas: Overall survival depending on p16 expression.** The absence of p16 expression was negatively related to prognosis; the median OS in the absence of p16 expression was 9.03 years (95% CI 3.6–14.47) versus 13.35 years (95% CI 9.36–17.35) for p16 expression (*P* < 0.05) (*N* = 71). Kaplan–Meier curves univariate survival analysis using comparisons between different groups using the Log-Rank test.

The absence of p16 expression was negatively related to prognosis and was statistically significant: the median OS in the absence of p16 was 9.03 years (95% CI 3.6–14.47) versus median OS with p16 expression of 13.35 years (95% CI 9.36–17.35); *P* = 0.023, HR = 0.41 (Fig. 2).

### P16 expression by IHC in patients with Grade 2 oligodendroglioma

**Figure 3:**
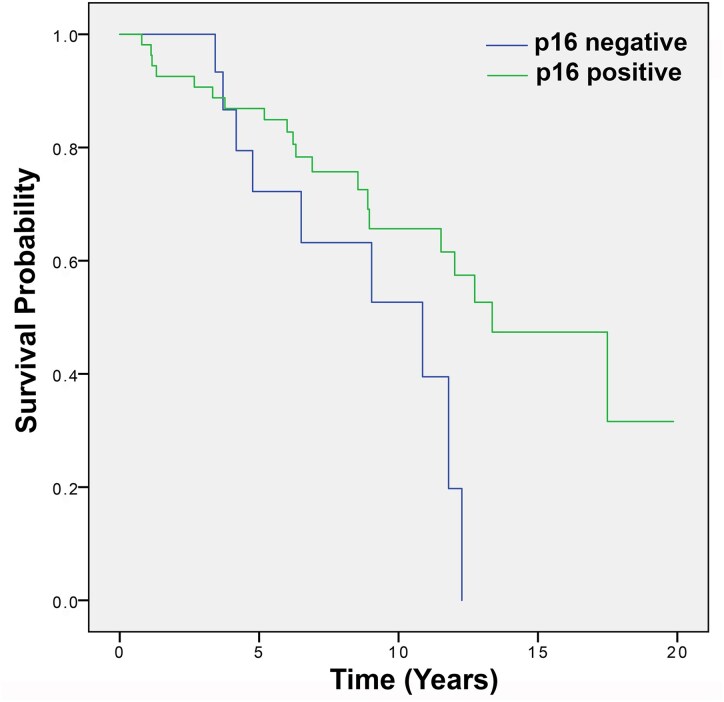
**Grade 2 oligodendrogliomas: overall survival depending on p16 expression.** The absence of p16 expression was observed in 16.3% cases of Grade 2 oligodendroglioma and was not significantly related to OS; median 10.86 years (95% CI 9.05–12.66) without p16 expression versus 13.35 years (95% CI 10.15–16.56) for p16 positive (*P* = 0.754) (*N* = 43). Kaplan–Meier curves univariate survival analysis using comparisons between different groups using the Log-Rank test.

p16 expression was analysed in 43 cases of Grade 2 oligodendroglioma. The absence of p16 expression was observed in 7 cases (16.3%). The absence of p16 expression was not significantly related to OS: median OS for those without p16 expression was 10.86 years (95% CI 9.05–12.66) versus 13.35 years (95% CI 10.15–16.56) for those expressing p16; *P* = 0.754 (Fig. 3).

### P16 expression by IHC in patients with Grade 3 oligodendroglioma

**Figure 4:**
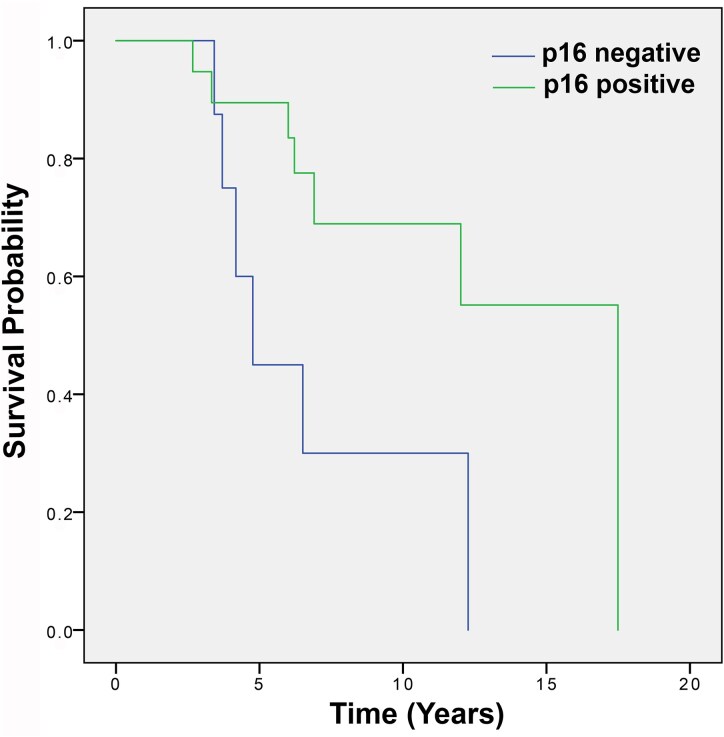
**Grade 3 oligodendrogliomas: overall survival depending on p16 expression.** p16 expression was absent in 32.1% cases of Grade 3 oligodendrogliomas and was negatively related to prognosis; median OS 4.76 years (95% CI 3.40–6.12) in the absence of p16 expression was versus 17.49 years (95% CI not reached) in the presence of p16 expression (*P* = <0.01) (*N* = 28). Kaplan–Meier curves univariate survival analysis using comparisons between different groups using the Log-Rank test.

Expression of p16 was analysed in 28 cases of Grade 3 oligodendrogliomas. p16 expression was absent in 9 cases (32.1%). The absence of p16 expression was negatively related to prognosis and was statistically significant: median OS in the absence of p16 expression was 4.76 years (95% CI 3.40–6.12) versus 17.49 years (95% CI not reached) in the presence of p16 expression; *P* = 0.010 (Fig. 4).

### Multivariate analysis and absence of p16 expression

A multivariate analysis was also performed including factors such as the extent of surgery, tumour grade, administration of adjuvant treatment and absence of p16 expression. Only the absence of p16 expression had a prognostic value that reached statistical significance, *P* = 0.034.

### P16 expression and microvascular proliferation, necrosis, and contrast uptake on MRI in Grade 3 oligodendroglioma

An analysis was done to find a possible correlation between p16 expression loss and microvascular proliferation, necrosis and contrast uptake on MRI in Grade 3 oligodendrogliomas.

The correlation between the loss of p16 expression and the presence of microvascular proliferation in Grade 3 oligodendrogliomas was analysed and no statistically significant relationship was found; Chi-square 0.802. We observed that a total of 23/32 cases (85%) of Grade 3 oligodendrogliomas had microvascular proliferation. Moreover, in 9 cases, there was information on both, microvascular proliferation and p16 expression, and in 8 of these cases (88%), in 8 cases with microvascular proliferation (8 cases) a loss of 16 expression was observed, and only in 1 case no microvascular proliferation observed.

Regarding necrosis, no statistically significant relationship was found between the loss of p16 expression (Chi-square 0.175) and the presence of necrosis.

Considering contrast enhancement on MRI in anaplastic oligodendrogliomas (where data were available), no statistically significant correlation with loss of p16 expression was found (Chi-square 0.668).

### Cyclin D1 expression

Cyclin D1 expression was analysed in 72 cases of the reclassified cohort. The expression was 0% in 40 cases (55.5%), 0–<10% in 18 cases (25%), 10–<25% in 8 cases (11.1%), 25–<50% in 5 cases (6.94%) and 50–<75% expression in 1 case (1.4%).

In Grade 2 oligodendrogliomas, there was no expression in 30 cases (71.4%) and in Grade 3 oligodendrogliomas there was no expression in 9 cases (32.1%).

### Expression of cyclin D1 and its prognostic impact

The prognostic value of cyclin D1 was analysed. High expression was considered when there was >25% expression. For the whole cohort, high cyclin D1 expression was found in 6 cases (6.6%), 2 cases (3.4%) of Grade 2 oligodendrogliomas and 4 cases (12.5%) of Grade 3 oligodendrogliomas.

**Figure 5:**
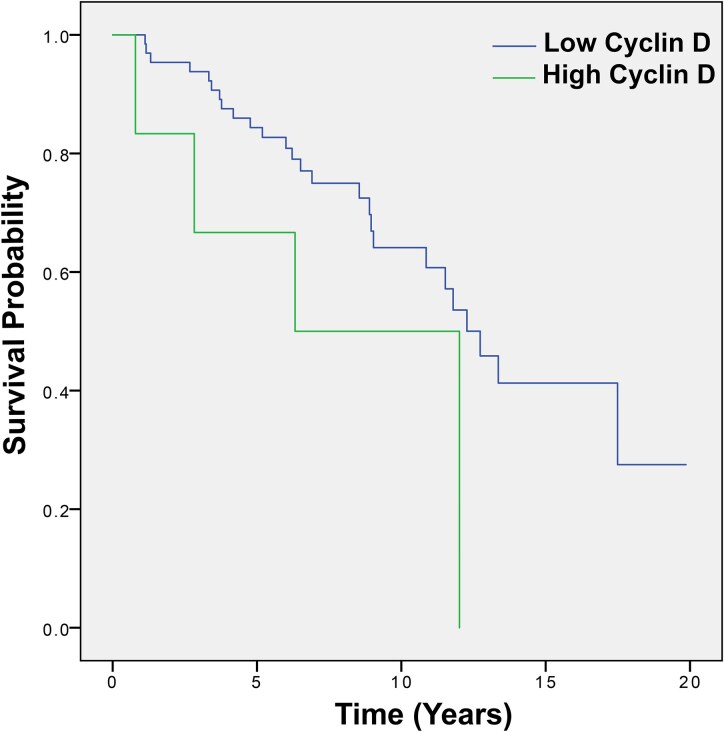
**Overall survival depending on cyclin D1 expression.** High D1 cyclin expression (>25%) was negatively related to prognosis; the median overall survival for low expression cyclin D1 was 12.73 years (95% CI 10.79–14.67) versus 6.31 years (95% CI 0.80–11.83) for high expression; *P* = 0.054 (*N* = 72). Kaplan–Meier curves univariate survival analysis using comparisons between different groups using the Log-Rank test.

There was a correlation between high expression and survival, which was at the limit of statistical significance for the whole cohort; the median overall survival for low expression cyclin D1 was 12.73 years (95% CI 10.79–14.67) versus 6.31 years (95% CI 0.80–11.83) for high expression; *P* = 0.054 (Fig. 5).

For Grade 2 oligodendrogliomas, high cyclin D1 expression was associated with significantly decreased median OS, for low expression was 12.73 years (95% CI 10.44–15.01) versus 0.78 years for high expression; *P* = <0.001.

There was no statistically significant difference for high cyclin D1 expression and overall survival in Grade 3 oligodendrogliomas 12.27 years for high D1 expression (95% CI 3.39–21.14) versus 12.01 years (no CI) for low expression (*P* = 0.786).

### Rb expression

Rb expression was analysed by immunohistochemistry in a total of 80 cases. Rb expression was found by IHC in 75 cases (93.8%) and 5 cases (6.2%) were negative. Of these 5 cases, 3 were Grade 2 and 2 were Grade 3 oligodendrogliomas. No prognostic impact has been found as a function of Rb expression.

## Discussion

In this reclassified cohort, for the total of patients analysed (Grades 2 and 3), IDH2 mutation was observed in approximately 10% of the cases. Other authors have found a somewhat lower percentage (around 5%) of IDH2 mutations.^10^

By tumour grade, in Grade 2 oligodendrogliomas, IDH2 mutations were found in 3% of cases in the EORTC 22033 study,^11^ and in Grade 3 oligodendrogliomas, in the RTOG 9402 study,^12^ 156 cases had a mutation, and an IDH2 mutation was found in 2 cases.

We found that the type of IDH1 or IDH2 mutation did not have a prognostic impact on survival.

A mutation in the TERT promoter was found in 88% of the cases and only 4.4% were wild type. The most frequent mutation was C228 (45–62%). These data were found in the overall cohort and also in Grade 2 and Grade 3 oligodendrogliomas. This is consistent with other published studies, that have confirmed this high rate of TERT mutation in this tumour. Also, in the EORTC 26951 study, TERT mutations were found in 98% of cases with codeletion.^13^

In our study, we found that neither the presence of TERT mutations nor the type of mutation has an impact on survival.

Analysing these data by tumour grade, there were no marked differences in the percentage of IDH2 nor TERT mutations between Grade 2 and Grade 3 oligodendrogliomas.

### Discussion of cyclin pathway data in the reclassified cohort

In our acknowledgment, our study is the first work analysing de cyclin pathway in oligodendrogliomas classified based on the current 2021 WHO classification, including molecular parameters (IDH mutation and 1p/19q codeletion). Many of the previous studies had analysed a series of cases of oligodendrogliomas solely diagnosed by histopathological criteria. These studies were published in the 1990s and early 2000s.

This is particularly important because molecular classification allows a more accurate selection of oligodendroglioma, excluding other types of tumour that could have oligodendroglial appearance.

### Discussion of p16 expression results

In our series, the absence of p16 expression was found in both Grade 2 and Grade 3 oligodendrogliomas: in 16% of Grade 2 and 32.1% of Grade 3. Moreover, this loss of expression in our series was associated with a statistically significant worse overall survival in Grade 3 oligodendrogliomas (with median OS of 4.76 years versus 17.49 years in absence versus presence of p16 expression) and also in the set of cases of Grade 2 and Grade 3, where the median survival was 9.03–13.35 years in the absence versus presence of p16, respectively, with an HR of 0.41. In the analysis of Grade 2 oligodendrogliomas, the difference did not reach statistical significance (10.86 years versus 13.35 years). These data highlight the presence of loss of p16 expression in these tumours, as well as its prognostic significance.

Other previous studies have analysed the importance of the cyclin pathway in these tumours. One of the aspects to consider is that the different studies have used different approaches, analysing either chromosome 9 loss or CDKN2A deletion or p16 expression, between which there is an interrelation, given that the CDKN2A gene is located on 9p21, and synthesizes p16 protein. p16 acts to inhibit the cell cycle proliferation and therefore it is a tumour suppressor gene.

From studies performed in the 1990s, Bigner *et al*.^14^ analysed 9p loss and/or deletion of the CDKN2A in 55 oligodendrogliomas and found a loss of 9p or deletion of CDKN2A in 42% of Grade 3 cases versus 14% in Grade 2. In concordance with this, our study showed a loss of p16 expression in 32% and 16%, respectively.

These data reflect the importance that CDKN2A/p16 may have in oligodendroglial tumours and the progression from Grade 2 to Grade 3 oligodendrogliomas.

Other authors, such as Cairncross *et al.*,^15^ described that CDKN2A deletions occurred mainly in tumours with intact 1p/19q. This may indicate that CDKN2A is involved only in tumours of another histological type such as astrocytes (and not in oligodendroglioma). However, our data, performed on a series with cases of oligodendrogliomas with mutated IDH and 1p/19q loss corroborate the data found by Bigner *et al.*, where an association of 9p/CDKN2A loss and oligodendrogliomas with loss of 1p/19q was observed.

More recent studies have also found the involvement of loss of 9p, CDKN2A deletion, or loss of p16 expression in oligodendroglial tumours. Appay *et al.*^16^ found a homozygous deletion in CDKN2A in 33% of the cases of Grade 3 oligodendrogliomas in a series of 483 cases with mutated IDH and loss of 1p/19q.

In Grade 2 oligodendroglioma, the data are more controversial, some authors such as Bigner *et al.*^14^ found loss of chromosome 9 in 21% of Grade 3 cases but none in low-grade cases. Also, in the study by Appay *et al*.^16^ there were no cases of homozygous deletion of CDKN2A in Grade 2 gliomas (codeleted or not, in a total of 40 cases).

In our study, we found a loss of p16 expression in 16% of cases.

The CDKN2A deletion data in our series, which is planned to be further developed, will better confirm the data found with p16.

Another issue that has been investigated in other studies is the correlation between p16 loss and histological findings (microvascular proliferation, necrosis, or RMI uptake).

In our cohort, a statistically significant correlation between p16 loss and microvascular proliferation was not found. There seems to be an association between the loss of p16 expression and the presence of microvascular proliferation, although statistical significance was not reached, as has been reported in other papers.^17^

Godfraind *et al.* found an association between the loss of chromosome 9 and the presence of microvascular proliferation and necrosis in Grade 3 oligodendrogliomas. In their series, 8 out of 9 patients with anaplastic oligodendrogliomas with microvascular proliferation also had a deletion of 9p.^17^

Figarella-Branger *et al.*^18^ reviewed 203 cases of anaplastic oligodendrogliomas and found that microvascular proliferation was associated with the loss of chromosome 9.

Regarding necrosis, we did not find a statistically significant relationship with the loss of p16 expression (Chi-square 0.175).

In our cohort, there was no statistically significant correlation between MRI contrast uptake and the loss of 16 expressions in anaplastic oligodendrogliomas (Chi-square 0.668). However, Reyes-Botero *et al.*^19^ reviewed 50 cases of anaplastic oligodendrogliomas and found that contrast-enhanced MRI imaging was significantly associated with loss of 9p and CDKN2A. This discrepancy from other studies may be related to the small number of patients analysed in our sample.

Another issue that has been analysed in our cohort is the prognostic impact of p16 loss. Indeed, the loss of p16 was significantly associated with a worse prognosis, most clearly in Grade 3 oligodendrogliomas.

Several studies have emphasized the prognostic value of loss of chromosome 9, deletion of CDKN2A, or p16 expression loss.

Miettinen *et al.*^20^ also analysed the prognostic value of CDKN2A in 42 oligodendrogliomas (not selected by current molecular criteria) and found that CDKN2A deletion/loss of p16 expression was associated with a worse prognosis in oligodendrogliomas.

Appay *et al.* reviewed 483 cases of anaplastic oligodendroglioma IDH mutated and 1p/19q codeleted, and found that the deletion of CDKN2A was an adverse poor prognostic factor. Furthermore, they found that CDKN2A deletion was a prognostic factor in both non-codeleted (they analysed 428 cases) and codeleted (483 cases) cases.^16^ Another very interesting aspect of the study published by Appay *et al.*, is that CDKN2A deletion appears to be the first risk stratification factor (better than the criteria of microvascular proliferation and necrosis). The presence of these three features was only relevant in the absence of CDKN2A deletion. The authors concluded that CDKN2A may be of great interest for the grading and classifying of these tumours. Our study is in line with these data, as p16 proves to be a prognostic marker in oligodendrogliomas. In our series, the multivariate analysis including the extent of surgery, adjuvant treatment, tumour grade and presence or absence of loss of p16 expression showed that the only significant prognostic factor observed was the loss of p16 expression (*P* = 0.034). These results corroborate those of Appay *et al.*, they also performed a multivariate analysis taking into account age, the extent of surgery, adjuvant treatment, and microvascular proliferation and necrosis; CDKN2A deletion remained as an independent prognostic factor.

Other studies also selected cases by current criteria. The study of Michaud *et al.*^21^ analysed the expression of p16 and also the deletion of 9p in a series of oligodendrogliomas with IDH mutation and 1p/19q codeletion. They found 9p deletion in 55% of Grade 3 oligodendrogliomas and no deletion of 9p in Grade 2 oligodendrogliomas. The deletion of 9p was associated with worse survival. They also analysed p16 expression by IHC, and the loss of p16 expression was associated with worse survival in Grade 3 oligodendrogliomas with 9p deletion, but not in patients with intact 9p or in those with Grade 2. For cases with 9p deletion and loss of p16, the survival was 2.33 years, and for those who maintained p16 expression 4.5 years, which was statistically significant. In our series, the differences were clear in Grade 3 oligodendroglioma, 4.76 versus 17.49 years, and were statistically significant. The authors consider in the discussion that there is a decrease in p16 expression in patients with 9p deletion and this leads to loss of CDK4/6 inhibition and increased cell proliferation. However, in the absence of deletion of 9p, the unfavourable evolution of these cases does not occur through the loss of p16 expression but by other mechanisms, and in these cases, it could lead to an overexpression of p16 that would be activated as a tumour suppressor (but inefficiently) to compensate for other activated oncogenic pathways.

Loss of chromosome 9 has also been previously studied by Alentorn *et al.*, who analysed 216 patients with oligodendroglial tumours with loss of 1p/19q. Chromosomal loss of 9p21.3 was detected in 42% of cases and was associated with a worse prognosis (both in PFS and OS).^22^

These data imply that chromosome 9 loss/CDKN2A deletion/loss of p16 expression could be used to categorize the prognosis of these patients and may even have value over and above tumour grade classification according to the data found in our series.

Indeed, the WHO 2021 classification has introduced CDKN2A deletion for the grading system of astrocytoma tumours. This classification includes astrocytomas, IDH1 mutated, WHO Grade 4 defined by either astrocytoma with IDH mutated, with microvascular proliferation or necrosis or CDKN2A/B deletion or any combination of these features.

As we have just described for astrocyte-type tumours, the data from our series support the value of the p16 expression loss as a molecular mechanism that can also allow risk classification in oligodendrogliomas.

This may have clear implications for the criteria for recommending adjuvant treatment in clinical practice, as lower-risk patients would be better candidates for follow-up, rather than more aggressive treatment with chemo- and radiotherapy. Prospective studies with larger numbers of patients are needed to confirm these data.

The implication of p16 expression in the response to therapy in gliomas, and specifically in oligodendrogliomas, is a field still to be explored. However, in other types of cancers, studies are already beginning to be published, such as in the case of non-small-cell lung cancer where the loss-of-function mutation of CDKN2A has been identified as a potential sensitivity to anti-PD-1 treatment *in vitro* and *in vivo*.^23^

One issue to consider is whether loss of p16 expression is a good surrogate marker of CDKN2A deletion and or loss of chromosome 9. We found few studies aimed at answering this question in glial tumours. Purkait *et al.*^24^ searched for a correlation between CDKN2A deletion and p16 protein expression in glioblastomas. They considered p16 positive when at least 1% of tumour cells expressed p16 and found that the correlation was highly significant: 85% of patients with CDKN2A deletion had a loss of p16 expression. Loss of p16 had a high sensitivity (81.5%) and a very high negative predictive value (84.8%). Bigner *et al.* found in their series of anaplastic oligodendrogliomas, 10 cases with a loss of 9p, of which 6 cases also had a loss of CDKN2A. Other series such as Rao *et al.* and Bruns *et al.* also found a good correlation between loss of p16 expression and CDKN2A deletion. In both studies, all cases with homozygous deletion of CDKN2A had a loss of p16 expression.^25,26^

Nevertheless, other studies have not found a good correlation between p16 expression by IHC and the detection of CDKN2A deletion. The authors pointed out that, in any case, the basal nuclear expression they found was too low in most samples to allow a definitive detection of loss of expression.^27,28^

Although we have not determined CDKN2A deletion in our series, we consider that this determination of CDKN2A could provide also relevant data.

### Discussion of cyclin D1 results

The expression of cyclin D1 was also analysed. Cyclin D1 expression was present in both Grade 2 and Grade 3 oligodendrogliomas, with a higher expression of cyclin D1 in Grade 3 oligodendrogliomas. The absence of expression in Grade 2 oligodendrogliomas was 71%, and the absence of expression in Grade 3 oligodendrogliomas was 32%.

Fiano *et al.*^29^ found that cyclin D1 levels increased with the degree of anaplasia, with a linear correlation with MIB1 index labelling. Other studies in animal models suggest that cyclin D1 and CDK4 play an important role in glioma tumour development and the tumour microenvironment influencing tumour growth, the loss of CDK4 prevented tumour development and the loss of cyclin D1 prevented progression to higher grades of malignancy.^30^

The prognostic value of cyclin D1 expression was analysed, high expression was considered when the expression was above 25% (6 cases). There was a correlation between overexpression and a worse prognosis, both in the series as a whole and in Grade 2 oligodendrogliomas (median 6.31 years versus 12.73 years). Michaud *et al*.^21^ also found this correlation between D1 overexpression and a worse prognosis.

### Discussion of Rb results

We analysed the expression of Rb by immunohistochemistry. A total of 81 cases were analysed and no expression was found by IHC only in 5 cases of the total (5.5%). Of these 5 cases, 3 were Grade 2 oligodendrogliomas and 2 were Grade 3 oligodendrogliomas. It was not possible to define a specific molecular pattern in these 5 cases, all had IDH mutation and deletion of 1p/19q, all had TERT mutation, p16 was expressed in 3 of them, Cyclin D1 expression was only found in one of these five cases. Our series corroborates the low frequency of the loss of Rb expression. Appay *et al.*^16^ did not detect any case of anaplastic oligodendroglioma with homozygous deletion of Rb1.

This is a retrospective analysis, with the biases intrinsic to this type of study.

Therefore, the limitations of the study are related to the retrospective series and the small sample size. On the other hand, the sample is heterogeneous in terms of prognosis and treatments received, both surgical and adjuvant chemo- and radiotherapy. In fact, 38% of the cases had not received any adjuvant treatment.

To understand the impact of this heterogeneity, a multivariate analysis was performed, including the extent of surgery, whether or not adjuvant treatment had been administered and tumour grade, only the absence of p16 expression was found to have a prognostic value.

However, according to these results, p16 could be a useful biomarker either for prognosis or therapy.

CDK4/6 inhibitors such as palbociclib, ribociclib and abemaciclib have received FDA approval for patients with HR^+^/HER2^−^ breast cancer, becoming the standard treatment for this group of patients.^31^

In the case of gliomas and particularly oligodendroglioma patients, a multicenter, open-label, Phase II trial was conducted evaluating the efficacy and safety of palbociclib in oligodendroglioma patients who progressed on radiotherapy and chemotherapy with histologically and molecularly confirmed Grade 3 oligodendroglioma.^32^ However, the study was stopped early owing to the lack of efficacy, with 74% of evaluable patients progressing within 6 months, so it was concluded that palbociclib did not show favourable efficacy against recurrent oligodendroglioma. It should be noted that based on the results of this study, a future clinical trial to study the response to this treatment in oligodendroglioma patients based on p16 expression would be necessary to exclude the real therapeutic benefit.

## Conclusions

In this cohort of oligodendroglial tumours classified by the WHO 2021 criteria, there was approximately 10% of IDH2 mutation and almost 90% of TERT mutation. The loss of p16 expression is associated with a poor prognosis, particularly in Grade 3 oligodendrogliomas.

## Data Availability

The data that support the findings of this study are available from the corresponding author upon reasonable request.
